# A Quality Improvement Intervention Reduces the Time to Administration of Stat Medications

**DOI:** 10.1097/pq9.0000000000000021

**Published:** 2017-04-17

**Authors:** Gigimol Stephen, Dane Moran, Joan Broderick, Hanan A. Shaikh, Megan M. Tschudy, Cheryl Connors, Tammy Williams, Julius C. Pham

**Affiliations:** From the * Johns Hopkins Aramco Healthcare, Dhahran, Saudi Arabia; † Johns Hopkins Medicine Armstrong Institute for Patient Safety and Quality, Baltimore, Md.; ‡ Johns Hopkins University School of Medicine, Baltimore, Md.; and § University of Hawaii School of Medicine, Honolulu, Hawaii.

## Abstract

**Introduction:**

The delivery of urgent (“stat”) medications to hospitalized children is important for safe quality care. The goal of this study was to evaluate the effect of a set of interventions on the percentage of stat medications administered within 30 minutes of ordering.

**Methods:**

A pre–post study in 2 pediatric units (36 beds) in a private hospital in Saudi Arabia between January 2015 and September 2016. Interventions included structured communication requirements, introduction of a dedicated electronic inbox for stat medication orders sent by nurses to the pharmacy, and the use of a pink envelope for the delivery of stat medications. A multivariate logistic regression model was used to model percentage of medications administered within goal.

**Results:**

Three hundred four stat orders met inclusion criteria. The proportion of orders meeting the 30-minute goal increased from a mean of 20% to a mean of 49% after the interventions (*P* < 0.001). In the final month of the study, compliance reached a peak of 67%. The mean turnaround time from ordering to the administration of the medication decreased from 59.7 to 40.7 minutes (*P* < 0.001). On multivariate analysis, medication type and unit-based availability of medications were statistically significant predictors of turnaround time. The odds of compliance being achieved was 0.3 times less if the medication was not available on the unit.

**Conclusions:**

A set of interventions significantly increased the percentage of stat medications delivered within 30 minutes.

## Abstract 2

Supplemental Digital Content is available in the text.

## INTRODUCTION

To provide quality care to patients, time is often of the essence.^1^ The term “stat,” which comes from the Latin “statim,” meaning immediately,^2^ is designed to give priority to orders that are needed most quickly. Generally speaking, a stat medication order should be administered within 30 minutes of the time it is ordered (turnaround time).^3^ Despite this informal standard, many hospitals struggle with delivering stat medications consistently under 30 minutes.

Timely delivery of stat medications is important for high-quality care. Mortality increases in patients with sepsis every hour that antibiotics are delayed,^4^ but unfortunately delays in antibiotic administration are a common occurrence.^4–7^ Furthermore, for children in status epilepticus, delayed administration of antiepileptics results in more prolonged seizures^8^ and lower antiepileptic medication responsiveness.^9–12^ Many reasons could explain why delays in the administration of urgent medications occur including the time taken to prepare the medication, deliver the medication to the unit, and administer the medication; insufficient staffing; poor communication; and lack of prioritization of stat medications. We believe that it is important for hospitals to identify and address any modifiable factors that could contribute to delays in the administration of these often life-saving medications.

We performed this study to evaluate the effect of a set of interventions on the proportion of stat medications that were delivered within 30 minutes to pediatric inpatients.

## METHODS

This study was approved by the Institutional Review Board at Johns Hopkins Aramco Healthcare. This pre–post interventional study was conducted in 2 general pediatrics units (36 beds total) at Johns Hopkins Aramco Healthcare. This hospital is a 350-bed private hospital that provides healthcare to Aramco employees and their families and is a Joint Venture between Saudi Aramco and Johns Hopkins Medicine. All patients aged 14 or younger are admitted to 1 of the 2 pediatric units.

### Patient Population

Patients admitted to the pediatric units aged 0–14 years were included in the study.

### Stat Orders

All stat orders were retrospectively reviewed by the quality improvement (QI) core team members, which comprised a QI specialist, a clinical nurse specialist, and the unit pharmacist. A physician was involved in the cases where further clarification was needed. Hospital policy was that stat orders were expected to be delivered within 30 minutes. Stat orders were excluded a priori if the patient was not able to receive the medication at the time of administration (eg, they were transferred to another unit, they were away for a procedure, or they required a stat laboratory before medication administration). Additionally, if multiple medications were ordered as stat at the same time, only the first medication administered was included; subsequent medications were excluded. For example, if 2 intravenous medications were ordered stat at the same time and the patient only had 1 intravenous line, then the second stat medication would need to be delayed.

### Measures

The primary outcome was the percentage of eligible stat medications administered within 30 minutes of ordering. The following data were also collected on each stat medication order: date and time of the order, medication ordered, whether the medication required pharmacy preparation or if it was available on the clinical unit, and the time from ordering to administration (turnaround time).

Turnaround time was broken down into discrete segments: ordered to scanned, scanned to processed, and processed to administered. Time ordered was the time the physician placed the medication order. Time scanned was the time when the nurse scanned the order into the computer system for the pharmacy to view. Time processed was the time when the pharmacist activated the order in the pharmacy. Time administered was the time when the patient received the medication.

The medications ordered were grouped into the following categories: antibiotics, emergency respiratory medications (including albuterol, steroids, antihistamines, and epinephrine), pain medications (including acetaminophen, non-steroidal anti-inflammatory, and opioids), seizure medications (including benzodiazepines, barbiturates, carbamazepine, and valproic acid), and other miscellaneous medications (eg, insulin, intravenous fluids, antiemetics). Time of medication ordered was grouped into 3 categories, based on shift times: 7:00 am to 3:00 pm, 3:00 to 11:00 pm, and 11:00 pm to 7:00 am.

In general, compliance data were collected monthly by a unit-based QI specialist from January 1, 2015, until September 30, 2016. The times that the medication was ordered, scanned, processed, and administered were available electronically. Further order details were retrieved from the electronic medical record. Compliance data were shared with the clinical teams, enabling a discussion on what the team was doing well and what could be improved. However, data were not collected during some months due to insufficient staff time (eg, February, March, and May 2015). Data collection was originally spread out over every 3 months beginning in September 2015 due to the time-intensive nature of collecting the data, but monthly collection was resumed in June 2016 because it was observed that turnaround time was steadily declining during periods when the data were collected less frequently.

### Process and Barriers

**Fig. 1.**
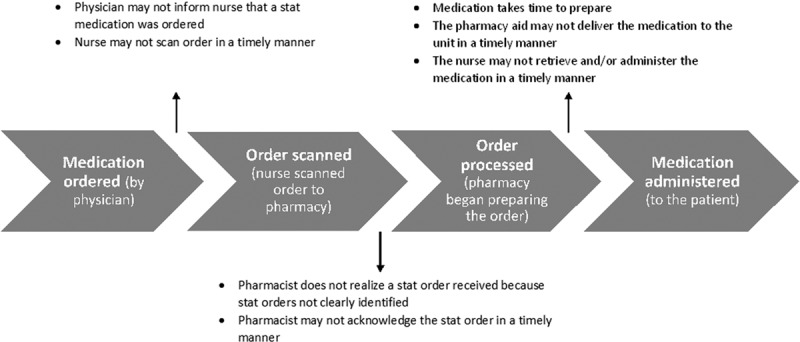
Simplified process map of medication administration from time of order, along with identifiable, modifiable barriers.

A team of nurses, physicians, and pharmacists was assembled to follow a Stat order through each step from ordering to administration. A process map was constructed of each step of stat medication ordering, preparation, and delivery, which was used to help identify barriers to timely medication administration (Fig. 1). A more detailed process map is also provided (Supplemental Digital content 1, http://links.lww.com/PQ9/A7). If the medication was available in the clinical unit, the nurse would be able to retrieve it after the order was processed in the pharmacy, and no additional preparation was required if the medication was available in the unit. If the medication was not available in the unit, then the pharmacy would prepare the medication and then deliver it to the unit for administration. Supplemental Digital Content 2, http://links.lww.com/PQ9/A8, lists the medications that were administered during the study period and whether they were available in the unit or came from the pharmacy.

### Interventions

Three interventions were employed to improve turnaround time. These interventions were started during the same period as part of an intervention set. First, improved communication systems between the physician and nurse and the nurse and pharmacist were established. The doctor was made responsible for alerting the charge nurse when (s)he ordered a stat medication. This practice was not done previously, which sometimes led to stat orders being delayed in the nursing station among the pile of other non-stat orders. The charge nurse became responsible for calling the pharmacy after (s)he had scanned the stat order to the pharmacy to ensure that the pharmacy was aware of the order. The doctor, nurse, and pharmacist held each other accountable for the compliance with these interventions. For example, if the charge nurse was not notified about the existence of a stat order from the physician, (s)he reminded the physician of the communication protocol.

Second, a stat-dedicated electronic inbox was created in the pharmacy so that stat orders were clearly identifiable to the pharmacist, as opposed to being mixed in with the other orders. Orders that were scanned by the nurse were delivered via e-mail to a stat-dedicated inbox for the pharmacist to review. Third, stat medications were placed in a pink envelope to be delivered to the unit. The pink envelope would alert the pharmacy aid, whose job was to deliver medications from the pharmacy to the unit, that the medications enclosed in the pink envelope were high priority, and thus should be delivered first. Although the first 2 interventions applied to all medications, the third was only applicable to medications coming from the pharmacy.

Members of the QI team were responsible for ensuring compliance with the interventions. Compliance with the interventions was tracked over time, and feedback was provided to the relevant parties monthly. The intervention set was implemented in April 2015—the period before this was considered preintervention and the period after this was considered postintervention.

### Analysis

Statistical analysis was performed using STATA 12 (StataCorp, College Station, Tex.). A *P* value of ≤0.05 was considered statistically significant. The chi-square test was performed to compare the proportion of orders meeting the 30-minute threshold. Linear and logistic regression were performed to compare turnaround times and proportions meeting stat time threshold, respectively, before and after the intervention. These were further stratified by type of medication and unit-based availability. Linear regression was performed to compare the turnaround time for each process segment before and after the intervention. Univariate logistic regression was performed to determine the influence of the following variables on meeting the turnaround time of 30 minutes: type of medication, administration time of day, and availability in the clinical unit. All predictor variables were deemed worthy of inclusion in the multivariate logistic regression model. The multivariate model was examined for collinearity and fit and was determined to be appropriately representative of our data.

## RESULTS

During the study period, 304 stat orders were included in the analysis. Twenty-one orders were excluded because they were ordered in conjunction with another stat medication, and 2 orders were excluded because the patient was off the unit. Of the orders that were included in the study, 46.4% were ordered during the day and 57.6% required pharmacy preparation. Antibiotics were the most commonly prescribed stat order (45.4%), followed by respiratory medications (20.4%; Table 1).

**Table 1.**
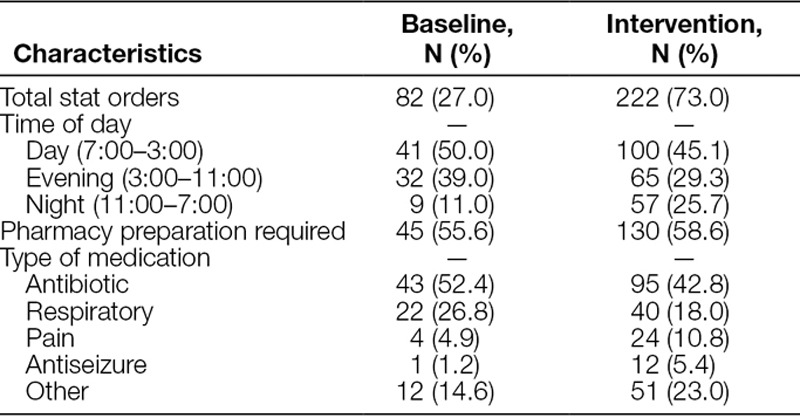
Study Population Characteristics

Compliance with the interventions was variable in the study. Compliance with the communication intervention was not formally assessed. The stat inbox intervention was implemented 100% of the time, and the pink envelope intervention was only used for 18 of 222 orders (8.1%).

**Fig. 2.**
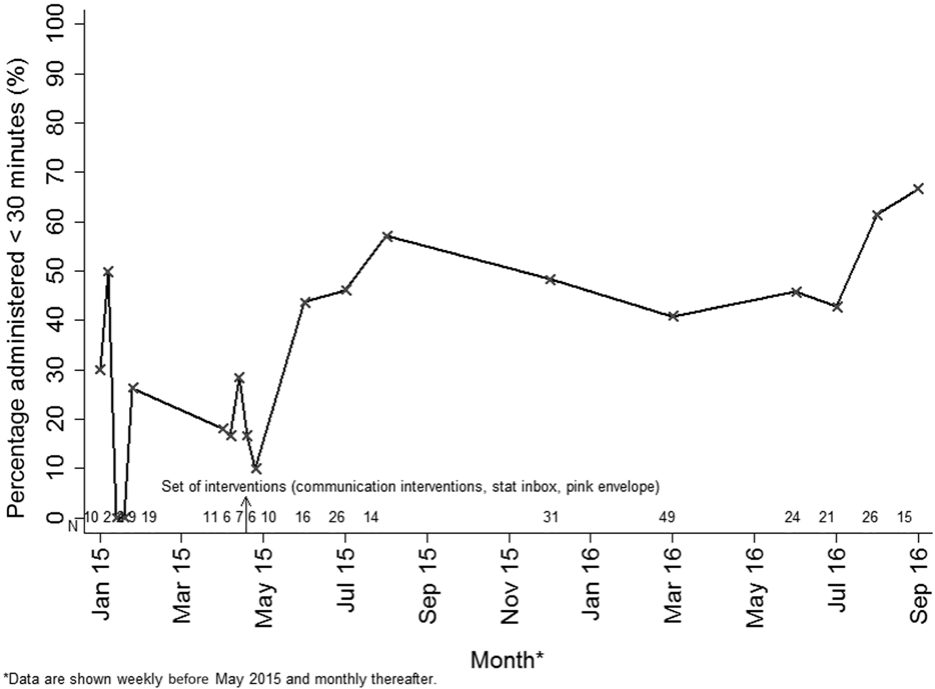
Percentage compliance to a turnaround time of 30 minutes for the receipt of a stat medication over the study period.

The proportion of orders with a turnaround time of <30 minutes increased following interventions (20% versus 49%; *P* < 0.001). Compliance with a turnaround time of <30 minutes tended to increase over time, with a peak compliance of 67% in September 2016 (Fig. 2). However, during the period when data were collected less frequently from September 2015 to June 2016, there was a steady decline in compliance that was observed. When monthly data collection resumed, compliance rapidly improved.

The mean turnaround time for the delivery of stat medications decreased from 59.7 (range, 10–150) to 40.7 (range, 4–200) minutes after the interventions (*P* < 0.001). Time decreased for medications that were prepared in pharmacy (70.7 versus 52.5 minutes; *P* < 0.001) and medications that were available in the clinical unit (45.8 versus 24.0 minutes; *P* < 0.001). After the interventions, there was a statistically significant improvement in the proportion of stat medications administered within 30 minutes for respiratory medications (27.3% versus 62.5%; *P* = 0.010) and pain medications (25.0% versus 95.8%; *P* = 0.006) but not for antibiotics (9.3% versus 19.0%; *P* = 0.160) or other miscellaneous medications (41.7% versus 68.6%; *P* = 0.089; Table 2).

**Table 2.**
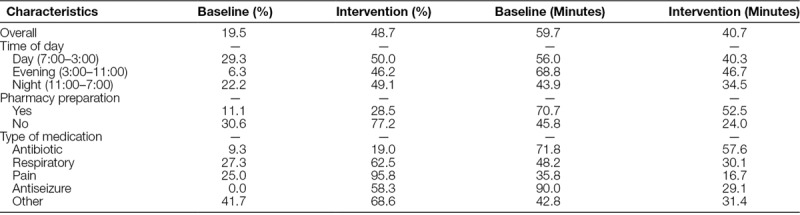
Proportion Meeting Turnaround Time Cutoff and Mean Turnaround Time

**Fig. 3.**
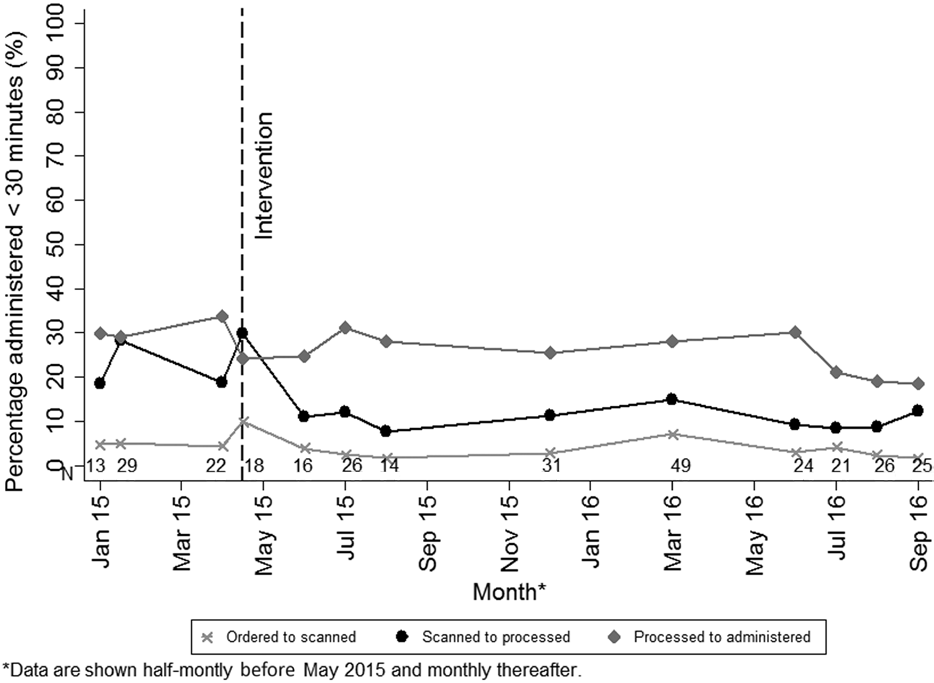
Mean turnaround time for each step from ordering to administration of a stat medication over the study period.

Among the various steps in the process from ordering to administering stat medications to patients, processing to administration took the most time, both before and after the intervention. The time between ordering and scanning took the least time (Fig. 3). A statistically significant reduction postintervention was seen for the time from scanned to processed (24.5 versus 11.2 minutes; *P* < 0.001). Nonstatistically significant reductions were experienced for the time from ordered to scanned (5.9 versus 3.8 minutes; *P* = 0.059) and processed to administered (29.3 versus 25.7 minutes; *P* = 0.218). The processed to administered time decreased for medicines available in the clinical unit (16.7 versus 13.1 minutes; *P* = 0.075) and for medicines that had to be retrieved from the pharmacy (38.7 versus 34.6 minutes; *P* = 0.344). The time taken for each step, stratified by whether the medication was available or not, is presented in Supplemental Digital Content 3, http://links.lww.com/PQ9/A9. The time between scanning and processing was the time that was reduced both for medications available in the unit and medications that were sent from the pharmacy.

On multivariate analysis, the odds of being compliant with a turnaround time of 30 minutes or less was 4.6 (1.7–8.0) times higher after the intervention (*P* < 0.001). The odds of being compliant was 0.30 (0.12–0.74) times lower if the medication was not available on the clinical unit (*P* = 0.009). The type of medication was a statistically significant predictor of compliance but time of day was not (Table 3).

**Table 3.**
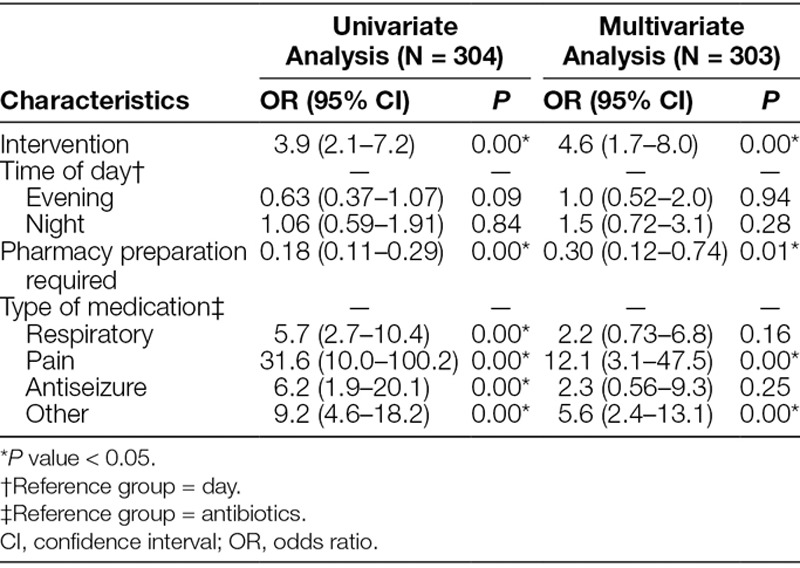
Univariate and Multivariate Analysis of Compliance with a Turnaround Target of 30 Minutes

## DISCUSSION

In this study, we found that a set of interventions, including structured communication requirements, an electronic stat inbox, and a pink envelope for medication delivery, increased the proportion of stat medications administered within 30 minutes from 20% at the start of the study period to 67% at the end of the study period.

Although the overuse of stat orders is well documented,^13–17^ there are relatively few published studies on the turnaround times hospitals can achieve for the administration of a stat medication.^18–20^ Our study demonstrates that a set of interventions can significantly improve turnaround times of stat medications, to the point where a majority are delivered under 30 minutes. We also found that turnaround times were significantly faster if the medication was available in the clinical unit, which is consistent with previously published findings.^21^

Although we increased the percentage of stat orders received on time, about one-third of them still were not administered within 30 minutes. Although we did not measure the clinical impact of this on patients, our experience suggests that there were no obvious adverse events. We question whether many of these orders needed to be given stat. Overuse of the stat designation for orders can lead to desensitization to the term and overload the system. Future efforts should be aimed at reviewing orders that truly need to be administered within 30 minutes and limiting the stat designation to those orders.

We found that several factors predicted a stat medication being administered in 30 minutes or less. Medications that were available in the clinical unit were clearly administered faster than if they had to come from the pharmacy. These medications were stored in electronic dispensing cabinets and did not require reconstitution. Regarding the type of medication, antibiotics were administered slowest among all the medication types. All antibiotics came from the pharmacy and required some preparation from the pharmacy before delivery. On the other hand, pain medications were administered the fastest, even after controlling for availability in the clinical unit. One possible explanation for this is that patients experiencing pain could have provided more frequent reminders to staff that they need their medication, which could have made the unit staff follow-up with the pharmacy in a more expeditious manner. The time of day did not have a significant influence on on-time stat medication delivery. Slower turnaround times were expected both in the evening (due to decreased staff and multiple shift changes) and at night (only 1 pharmacist to cover the whole hospital) compared with the day shift. However, during the day shift, there was a higher volume of orders and other competing priorities, which could explain why time of day did not play a role in determining stat compliance.

Two parts of the intervention appeared to be most critical. First, creating a separate inbox in the pharmacy appears to have been an important contributor to the substantial reduction in the time from receipt in pharmacy to processing. The inbox was a simple technological intervention that assisted with prioritization of orders in the pharmacy and enabled faster delivery times of both unit-based and pharmacy-based medications. Second, the improved communication between the physician and nursing staff at the time of order may have been responsible for the slight, statistically insignificant decrease in the time from order to scan. If the physician did not notify the nurse promptly, it was often the case that the nurse informed the physician of this delay. However, it is possible that other factors could have been responsible for this time decrease. Further studies would be required to determine whether this should be considered an evidence-based intervention. The intervention that we believe was the least successful was the introduction of the pink envelope because we observed that compliance with this intervention was very poor. Further, the time from processing to administration did not improve as much as we had hoped. Compliance with this intervention was likely so poor because it required extra work by the pharmacy aid and the nurse. Interestingly, when data collection was stretched out to every 3 months between August 2015 and June 2016, on-time stat med delivery decreased. However, once monthly data collection recommenced in June 2016, compliance improved again. It appears the more frequent monthly feedback reports, and the presence of consistent data collection served as an important reminder to the staff.

Because of this project, we have identified a few additional interventions that we could implement. First, hiring extra staff to deliver stat orders to the units could decrease the time from processing to administration. Second, given that medications that were available in the clinical units were administered more rapidly, stocking more of the most commonly used antibiotics (eg, ceftriaxone and vancomycin) in the unit could be effective in reducing antibiotic delays.

Our study has several limitations. First, all interventions in the set were implemented simultaneously; therefore, we cannot fully determine the effect of any single intervention. Second, data on patient outcomes were not available, and as such, it could not be determined whether reduced time to administration of stat medications resulted in improved outcomes. Having these data would help determine the ultimate effect on patients. Third, data were not collected on orders that were designated as stat but were later excluded. Fourth, we could not control for all the variables that might have influenced turnaround time for stat medications over the duration of the study period and thus cannot definitively demonstrate a causal relationship between our intervention and compliance. Fifth, we did not collect robust, quantitative data on the level of compliance to the communication intervention. Sixth, we did not quantitatively evaluate any unintended consequences of our intervention, such as delayed administration of non-stat medications due to limited resources and time constraints. However, from the monthly, interdisciplinary discussion sessions, no unintended consequences were reported by the staff. Other limitations include the irregularity with which the outcome data were collected and the short baseline period.

In summary, we found that a set of interventions increased the proportion of stat medications delivered within 30 minutes. Hospitals that have delays in their stat medications could consider adopting these interventions to decrease medication turnaround time.

## ACKNOWLEDGMENTS

The authors would like to thank Dr. Albert Wu for providing comments on the article, as well as Johns Hopkins Aramco Healthcare, the Armstrong Institute, and Johns Hopkins International for supporting this research.

## DISCLOSURE

The authors have no financial interest to declare in relation to the content of this article.
